# The Development of a Real-Time Artificial Intelligence System Using a Convolutional Neural Network for the Assessment of Infant Head Control

**DOI:** 10.31662/jmaj.2025-0499

**Published:** 2026-05-29

**Authors:** Akihiro Hosono, Katsura Ito, Mari Ichikawa

**Affiliations:** 1 Department of Public Health, Nagoya City University Graduate School of Medical Science and Medical School, Nagoya, Japan; 2 Nagoya City Health Center, Nagoya, Japan

**Keywords:** machine learning, artificial intelligence, infant head control, pull-to-sit test

## Abstract

**Introduction:**

To develop a real-time artificial intelligence (AI) system using a convolutional neural network for the assessment of infant head control (good or poor) during the traction response.

**Methods:**

Infants aged 3-6 months who received community-based group health checkups and whose guardians provided informed consent were enrolled in this study. Videos were recorded during the pull-to-sit test conducted by a pediatrician. The collected videos were divided into training and test datasets. Training data were extracted as image frames and labeled according to the pediatrician’s judgment. After supervised machine learning, the AI system was evaluated using the test dataset, and the sensitivity, specificity, and detection rate were calculated.

**Results:**

Altogether, 184 videos were collected and divided into the training (n = 110) and test (n = 74) datasets. The training and test datasets included 103 (93.6%) and 64 (86.5%) videos, respectively, showing infants with good head control. After machine learning, the screening AI system had sensitivity and specificity (95% confidence interval) values of 50.0% (18.7%-81.3%) and 98.4% (91.6%-100%), respectively. The detection rate was 100%.

**Conclusions:**

The proposed AI-based assessment system was able to identify good head control with high specificity during the pull-to-sit test but demonstrated limited sensitivity for detecting poor head control, suggesting a supportive rather than a standalone clinical role.

## Introduction

Head control is a very important milestone for infants after 4 months of age, and the presence of head lag is usually assessed when an infant lying supine is pulled into a sitting position (pull-to-sit test). Infants with head lag or delayed acquisition of head control may be suspected of having developmental disabilities such as cerebral palsy and autism spectrum disorder ^(1), (2)^. The Japanese local governments widely conduct community-based group health checkups for infants aged 3-5 months, wherein a pull-to-sit test is performed as a screening test ^(3)^. However, serious problems are still present and require solutions.

First, the number of pediatricians in Japan is not sufficient. Recently, a larger number of children require more medical care than before ^(4)^. Since April 2019, the Japanese Ministry of Health, Labor and Welfare introduced the “Work System Reform,” which included redress of long working hours, engagement of women and older adults in the workforce, and reduction of disparities. Moreover, most pediatricians work only in clinical areas. It has become much more difficult for pediatricians to perform health checkups for infants and children at local health centers, although, in the last two decades, the number of pediatricians has slightly increased and the number of children aged <14 years has decreased ^(5)^. Second, the criteria of good or poor head control have not been standardized in Japan. Every local government has provided a manual for conducting infant group health checkups, which includes the pull-to-sit test. However, these manuals and previous literature show slightly different criteria for evaluating good or poor head control ^(6), (7), (8)^. Moreover, the assessment of head control might depend on pediatricians’ skills. Third, providing follow-up care for infants with poor head control after health checkups is difficult because of the increasing number of double-income and nuclear families. Additionally, the coronavirus disease 2019 pandemic has accelerated the need for remote medical care systems. To address these problems, we developed a screening artificial intelligence (AI) system for the assessment of infant head control by using a convolutional neural network. This study aimed to develop and evaluate an AI-based system for assessing infant head control, with a focus on its potential role in supporting clinical assessment.

## Materials and Methods

### Data collection

Between August 2019 and March 2022, participants in a community-based group health checkup program for infants aged 3 months at Atsuta Health Center, Nagoya, Japan, were enrolled in the present study. The local government of Nagoya City conducts weekly and monthly community-based group health checkups at 16 local health centers for resident infants aged 3 months, 18 months, and 3 years, depending on the population. Each health center sends an invitation letter to the parents and holds group health checkups for each age group, 1-3 times a month. During the group health checkups of infants aged 3 months, the infants are generally aged >3 months but <6 months and undergo body measurements, medical examinations (including the pull-to-sit test), and nutrition and childcare consultations. Infants whose parents agreed to video recording during the 3-month checkup at Atsuta Health Center were included in the study.

Written informed consent was obtained from the infants’ parents prior to study participation. Infants whose parents declined video recording or were unable to understand Japanese were excluded from the study.

### Pull-to-sit test

With the infants in the supine position, both wrists were grasped, and the infants were gently pulled toward a sitting position. The response of the head (traction response) and the amount of head lag during the maneuver were assessed and recorded on video. The pull-to-sit test was performed by a single pediatrician certified by the Japan Pediatric Society (author K.I.) and independently reviewed by another pediatrician (author A.H.). In the present study, good head control (absence of head lag) was operationally defined as the condition in which the infant’s head was in an approximately midline position at least once during the pull-to-sit maneuver. This definition closely corresponds to the criteria described in the Nyuyoji Kenshin Manual, 7th edition ^(8)^, which is widely used in routine infant health checkups in Japan.

Videos of the pull-to-sit test were obtained from one or both sides of the body using a home video camera (HDR-CX470, SONY, Tokyo, Japan). All videos were recorded in the Advanced Video Coding High Definition (AVCHD) format at a frame rate of 60i and saved in the mp4 format for subsequent analysis.

### Data preparation and AI program construction

All system development was conducted by Neutral Co., Ltd. (Nagoya, Japan). To develop the screening AI system by using a convolutional neural network for the assessment of infant head control, we applied the fourth version of the You Only Look Once (YOLO, https://arxiv.org/abs/2004.10934) algorithm provided in 2020 by Alexey Bochkovsky. The YOLO series is widely used as one of the gold standard systems in real-time object detection, and the initial version was introduced in 2015 by Joseph Redmon.

**Figure 1.**
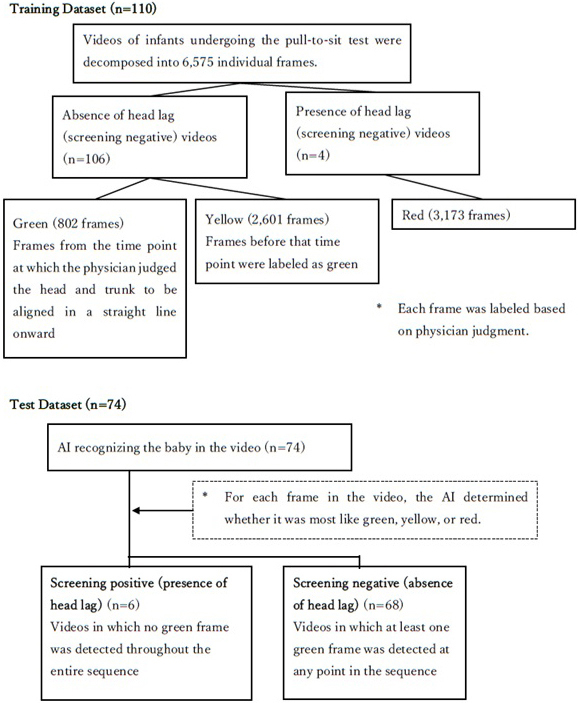
Decision flow diagram for the evaluation of training and test videos.

The collected videos were divided into the training and test datasets. Before supervised machine learning, training data images were extracted and labeled as follows: class 0: at the moment when physicians determined good head control (the head and trunk aligned in a straight line onward) during the test; class 1: between the beginning of the test and before class 0; and class 2: at the moment when physicians determined poor head control (the presence of head lag, Figure 1).

**Figure 2.**
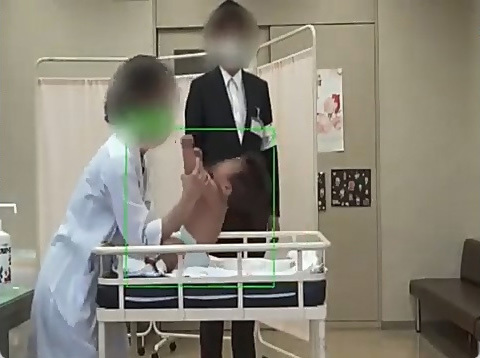
Absence of head lag. The infant is shown in a green bounding box.

**Figure 3.**
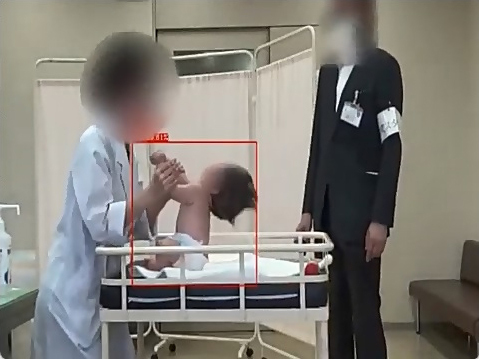
Presence of head lag. The infant is shown in a red bounding box.

To improve the detection rate of the screening AI system, some images were flipped or mirrored and adjusted in terms of exposure, saturation, hue, and angle. The screening AI system automatically detects subjects in the video, performs a real-time analysis of head control per frame, and displays videos with subject marking of bounding box color (green, yellow, and red for classes 0, 1, and 2, respectively). If a green bounding box is displayed at least once, the screening AI system detects good head control (Figures 2 and 3 ; Videos S1 and S2).

After supervised machine learning, we examined the performance of the AI system using the test data. The sensitivity, specificity, and detection rates were calculated as follows: sensitivity = detected number of correct poor head control / actual number of poor head control; specificity = detected number of correct good head control cases/ actual number of good head control cases; and detection rate = detected number of videos/number of videos in the test dataset.

We calculated the 95% confidence interval (CI) for sensitivity and specificity by using EZR on R Commander version 1.61 (Saitama Medical Center, Jichi Medical University, Saitama, Japan), which is a graphical user interface for R version 4.0,3 (The R Foundation for Statistical Computing, Vienna, Austria). Agreement between the physician’s judgment and the AI-based classification was evaluated using Cohen’s kappa coefficient.

## Results

From a total of 128 infants, 184 videos were collected and divided into 110 videos for the training dataset and 74 videos for the test dataset. Discrepancies were observed between the two reviewers in two videos (overall agreement, 99.2%), both of which were included in the test dataset. In these discrepant cases, the videos were conservatively classified as screening-positive (i.e., head lag-negative) based on clinical judgment, prioritizing patient safety. Given that one infant underwent a retest, the total number of videos included two retest videos recorded from both sides. The training dataset consisted of 103 videos showing infants with good head control and seven videos showing infants with poor head control, with 6,576 images extracted for supervised machine learning.

The testing dataset with 74 videos included 64 videos showing infants with good head control (86.5%). A total of five videos were labeled as the presence of head lag by the physician and the absence of head lag (screening false-negative) by the AI system, whereas one video was labeled the absence of head lag by the physician and the presence of head lag (screening false-positive) by the AI system (Table 1). Our screening AI system had sensitivity and specificity (95% CI) of 50.0% (18.7%-81.3%) and 98.4% (91.6%-100%), respectively, for detecting good head control among infants. The kappa value for Physician-AI agreement was 0.58 (95% CI: 0.26-0.90). The AI system detected all the infants in the videos (detection rate, 100%).

**Table 1.** Cross-Tabulation of Classifications Determined by Physicians using the Pull-to-Sit Test and by the AI System. Each cell denotes the number of videos.

		Physicians test		
		Screening positive (Presence of head lag)	Screening negative (Absence of head lag)	
AI test	Screening positive (Presence of head lag)	5	1	6
	Screening negative (Absence of head lag)	5	63	68
		10	64	74

## Discussion

In this study, we developed and evaluated a real-time AI system based on a convolutional neural network to assess infant head control during the pull-to-sit test. The results indicated that the proposed AI system achieved high specificity in identifying good head control (absence of head lag), whereas sensitivity for poor head control remained limited, suggesting a supportive rather than a standalone role in infant health assessments.

A key challenge in the evaluation of infant head control is the lack of fully standardized assessment criteria and the inherent subjectivity of clinical judgment. To address this issue, head control in the present study was evaluated by pediatricians using criteria consistent with commonly used clinical and neurological assessment scales, including the Premie-Neuro Examination, Head Control Scale, Dubowitz Neurological Examination, and Clinical Rating Scale for Head Control ^(9), (10), (11), (12), (13)^. The operational definition of the absence of head lag used in this study corresponded to thresholds indicating partial or complete head lifting in these established scales. The moderate Physician-AI agreement (κ = 0.58) suggests that the AI system approximated clinical judgment to a certain extent, while still leaving room for improvement. Analysis of misclassified cases provided important insights into the limitations of the current AI system. False-negative cases occurred when physicians noted the presence of head lag, but the AI system classified the case as showing the absence of head lag. These cases were attributable to inter-physician disagreement and misclassification by the AI model itself. A small number of false-positive cases were also identified, in which transient detections of appropriate head control contributed to misclassification. These findings suggest that transient transitions between green and red states may contribute to classification errors. Consequently, the criteria for tolerating such transitions in the final judgment remain insufficiently defined, highlighting the need for further refinement of the temporal decision rules in the algorithm.

Referring to the Organization for Economic Co-operation and Development (OECD) Reviews of Public Health ^(14)^, access to health checkups for preschool children is high in Japan, and nearly all infants and preschool children undergo health checkups, which are required by the government. In 2021, health checkups for infants aged 3-5 months were conducted by 99.5% of municipalities, with an infant participation rate of 95.5% ^(3), (15)^. Furthermore, the percentage of infants aged approximately 3 months who were enrolled in the study among those who underwent health checkups was 97.0% (128/132), which represents large-scale population-based data on health care for infants and preschool children. Given that the criteria of good head control may vary across municipalities, the use of a standardized AI-based assessment tool could contribute to harmonizing evaluation criteria and facilitating further studies on infant head control and related health care needs.

The present study also showed limited sensitivity for detecting poor head control, largely due to class imbalance in the training dataset. To reduce inter-examiner variability and establish fundamental technical feasibility, diagnostic judgments were intentionally based on assessments conducted by a single physician, which resulted in a limited number of poor head control cases. Consequently, the proposed AI system should not be regarded as a standalone screening tool at this stage. Instead, it may be more appropriately used as an auxiliary screening tool to support clinical assessments or as a training aid for beginners in developmental evaluation.

Despite these limitations, the present study successfully demonstrated the technical feasibility of real-time AI-based assessment of infant head control. Future studies should focus on the collection of multicenter data in clinical settings with a higher prevalence of head lag, such as developmental clinics and neonatal intensive care units, to enhance sensitivity and generalizability. Furthermore, future validation studies may benefit from adopting a noninferiority framework comparing AI-based assessments with physicians’ overall clinical developmental judgments, thereby better reflecting real-world clinical utility.

## Article Information

### Acknowledgments

Ito Akane, Tsutomu Yamahata, and Tatsuhiko Yamato, who work at Neutral Co., Ltd., were involved in visual AI training, including image extraction and labeling, in collaboration with Akihiro Hosono and the Nagoya City University Graduate School of Medical Science and Medical School. Keiko Mizutani, a research assistant in the Department of Public Health at Nagoya City University Graduate School of Medical Science and Medical School, was partially involved in collecting informed consents from the infants’ parents.

### Author Contributions

Conceptualization, data curation, project administration, methodology, resources, writing - review & editing, investigation: Akihiro Hosono. Investigation, formal analysis, writing - original draft: Mari Ichikawa. Investigation: Katsura Ito

### Conflicts of Interest

Akihiro Hosono currently holds a patent for the AI program developed as part of the present study. (Patent number 7099760). The other authors have no conflicts of interest to declare.

### IRB Approval Code and Name of the Institution

The study protocol was approved by the institutional review board of the Nagoya City University Graduate School of Medical Science and Medical School (approval number 60-19-0092) and the ethical review board of Nagoya City Public Health Research Institute (approval number 32). Written informed consent was obtained from the infants’ parents prior to study participation. All researchers involved in this study conducted the study in accordance with the Declaration of Helsinki, the Ethical Guidelines for Medical and Health Research Involving Human Subjects, and the study protocol.

### Data Set Availability

The datasets used and/or analyzed during the current study are available from the corresponding author on reasonable request.

## Supplement

Supplementary Material 1 Supplementary video 1. Absence of head lag. The baby is situated in a red bounding box, and its color changes to green.

Supplementary Material 2 Supplementary video 2. Presence of head lag. Until the end of pull-to-sit test, the bounding box remains red or yellow.
